# Supplementary material for: The HSF1–PARP13–PARP1 complex facilitates DNA repair and promotes mammary tumorigenesis
Source: Nat Commun. 2017 Nov 21;8:1638. doi: 10.1038/s41467-017-01807-7 (PMC5696371; doi:10.1038/s41467-017-01807-7)
Supplement: Supplementary file 1 — Supplementary Information [file 41467_2017_1807_MOESM1_ESM.pdf]

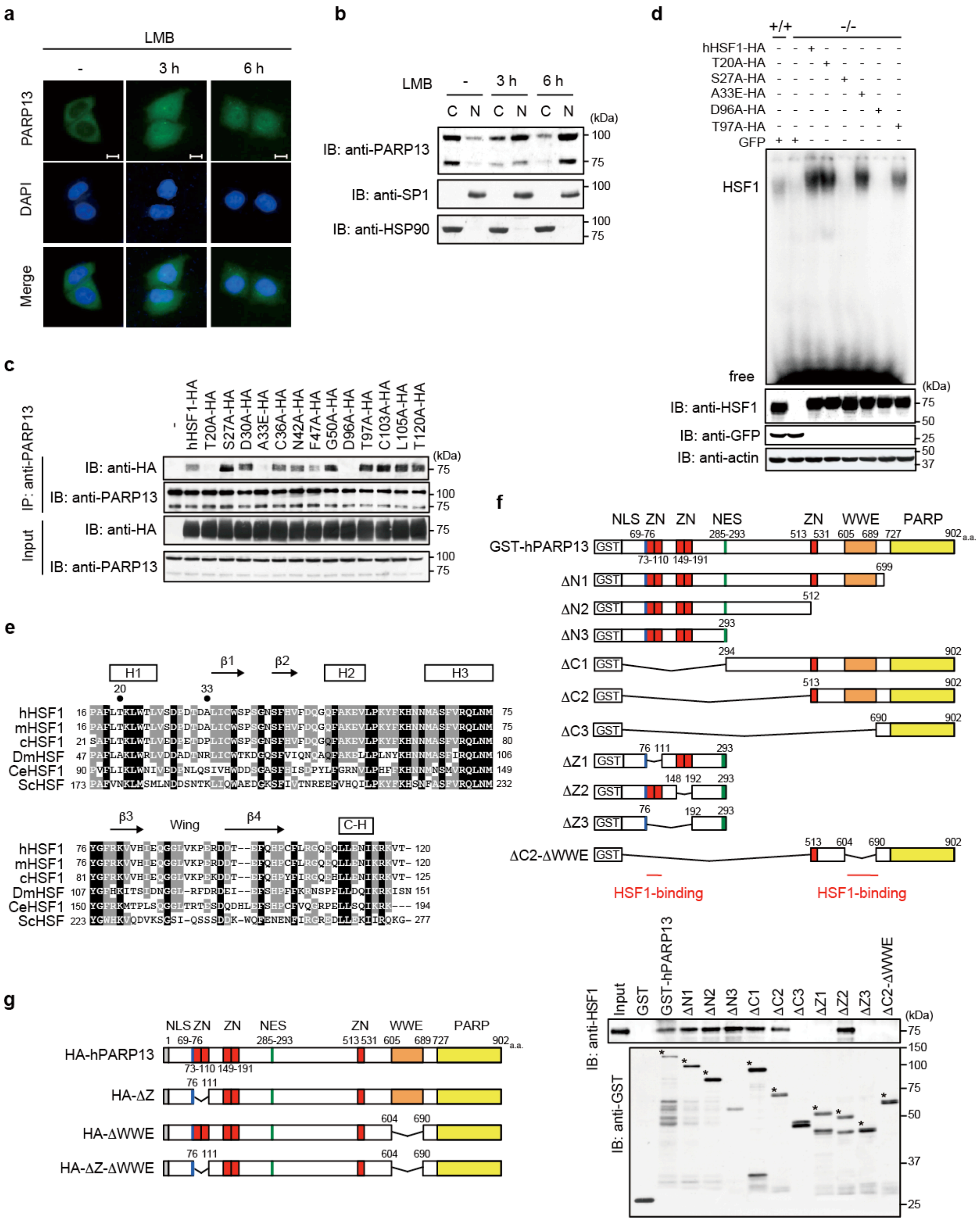
a
LMB
-
3 h
6 h
PARP13
DAPI
Merge
b
LMB
C N C N C N
(kDa)
IB: anti-PARP13
IB: anti-SP1
IB: anti-HSP90
100
75
c
IP: anti-PARP13
Input
IB: anti-HA
IB: anti-PARP13
hHSF1-HA
T20A-HA
S27A-HA
D30A-HA
A33E-HA
C36A-HA
N42A-HA
F47A-HA
G50A-HA
D96A-HA
T97A-HA
C103A-HA
L105A-HA
T120A-HA
d
+/+
-/-
hHSF1-HA
T20A-HA
S27A-HA
A33E-HA
D96A-HA
T97A-HA
GFP
HSF1
free
IB: anti-HSF1
IB: anti-GFP
IB: anti-actin
e
H1
β1
β2
H2
H3
β3
Wing
β4
C-H
hHSF1
mHSF1
cHSF1
DmHSF
CeHSF1
ScHSF
f
NLS ZN ZN NES ZN WWE PARP
GST-hPARP13
ΔN1
ΔN2
ΔN3
ΔC1
ΔC2
ΔC3
ΔZ1
ΔZ2
ΔZ3
ΔC2-ΔWWE
HSF1-binding
HSF1-binding
Input
GST
GST-hPARP13
IB: anti-HSF1
IB: anti-GST
g
NLS ZN ZN NES ZN WWE PARP
HA-hPARP13
HA-ΔZ
HA-ΔWWE
HA-ΔZ-ΔWWE

Supplementary Fig. 1. Interaction of HSF1 with PARP13

(**a** and **b**) HeLa cells were treated with LMB for the indicated periods. Fluorescence images were obtained (**a**), and cytoplasmic and nuclear fractions were subjected to immunoblotting (**b**). Scale bar, 10 μm. (**c**) HEK293 cells were transfected with wild-type and mutated hHSF1-HA, and subjected to PARP13 immunoprecipitation and immunoblotting. (**d**) Extracts of HSF1-null MEF cells (-/-), in which hHSF1 point mutants were expressed, were subjected to EMSA. GFP was expressed as a control. (**e**) Alignment of the amino acid sequences for the DNA-binding domains of human, mouse, and chicken HSF1 and *Drosophila melanogaster*, *C. elegans*, and *Saccharomyces cerevisiae* HSF. Thr20 and Ala33 in human HSF1 are indicated by dots. Predicted secondary structure including four α-helices (boxes H1 to H3 and C-H), four β-sheets (arrows β1 to β4), and a wing motif[35,36] is shown (Fig. 1e). (**f**) PARP13 directly interacted with HSF1 through two regions in vitro. GST-pull-down from mixtures of purified GST-fused hPARP13 mutants and hHSF1-His was performed, and proteins were subjected to immunoblotting. Asterisks indicate full-length GST-fusion proteins. HSF1-binding regions are indicated at the bottom of the diagram. ZN, zinc finger; WWE, conserved W, W and E residues; PARP, PARP domain; NLS, nuclear localization signal; NES, nuclear export signal. (**g**) Schematic representation of HA-hPARP13 mutants, which lack one of two HSF1-binding regions (ΔZ and ΔWWE) or both regions (ΔZ-ΔWWE) (Fig. 1f).

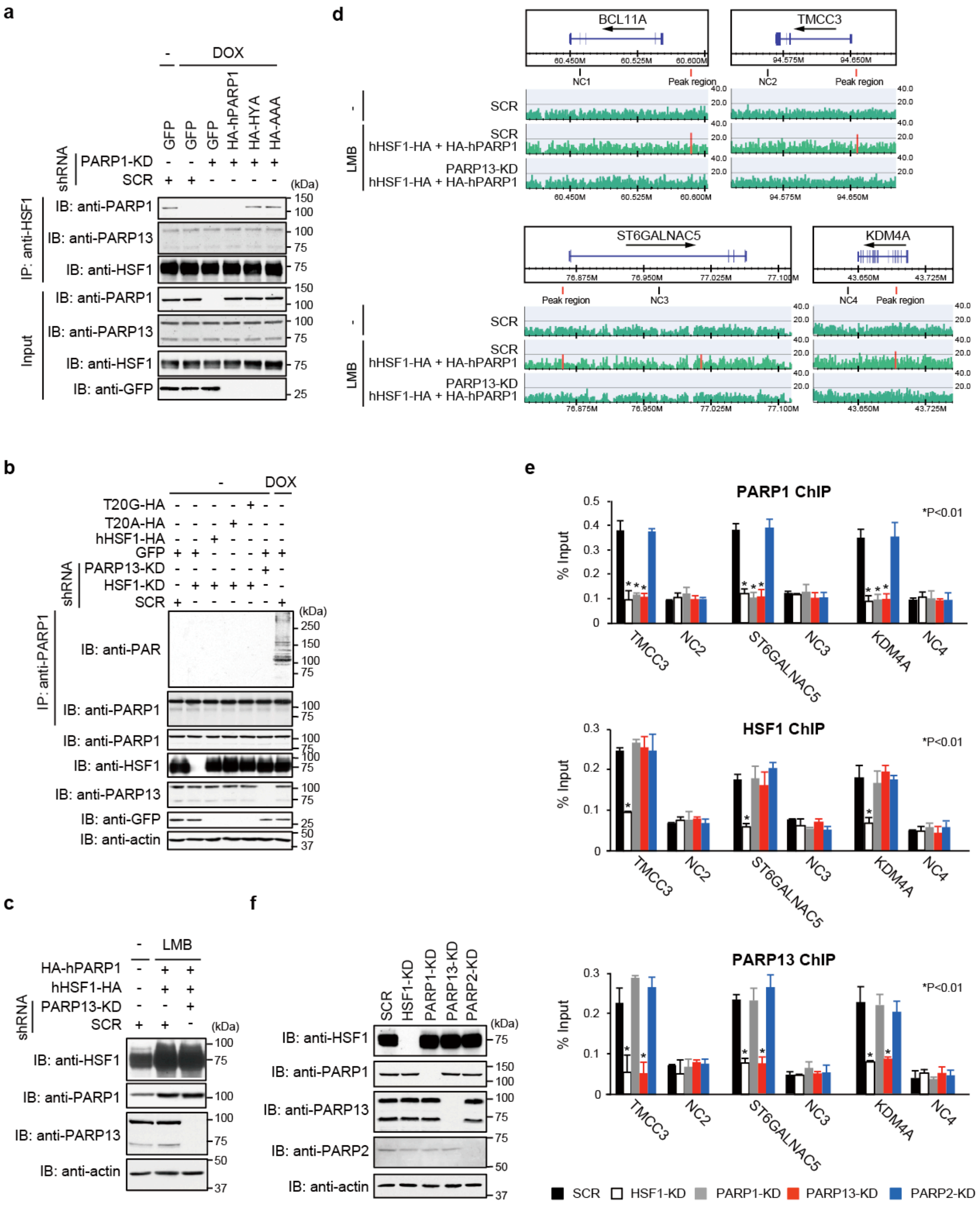
a
- DOX
GFP GFP GFP HA-hPARP1 HA-HYA HA-AAA
shRNA PARP1-KD - - + + + +
SCR + + - - - -
(kDa)
IP: anti-HSF1
IB: anti-PARP1
IB: anti-PARP13
IB: anti-HSF1
Input
IB: anti-PARP1
IB: anti-PARP13
IB: anti-HSF1
IB: anti-GFP
150 100 100 75 75 150 100 100 75 75 25
d
BCL11A
TMCC3
60.450M 60.525M 60.600M
94.575M 94.650M
NC1 Peak region NC2 Peak region
SCR
LMB
hHSF1-HA + HA-hPARP1
PARP13-KD
40.0 20.0
ST6GALNAC5
KDM4A
76.875M 76.950M 77.025M 77.100M
43.650M 43.725M
Peak region NC3 NC4 Peak region
b
- DOX
T20G-HA
T20A-HA
hHSF1-HA
GFP
shRNA PARP13-KD
HSF1-KD
SCR
(kDa)
IP: anti-PARP1
IB: anti-PAR
IB: anti-PARP1
IB: anti-PARP1
IB: anti-HSF1
IB: anti-PARP13
IB: anti-GFP
IB: anti-actin
250 150 100 75 100 75 100 75 100 75 100 75 25 50 37
e
PARP1 ChIP
HSF1 ChIP
PARP13 ChIP
% Input
*P<0.01
TMCC3 NC2 ST6GALNAC5 NC3 KDM4A NC4
SCR HSF1-KD PARP1-KD PARP13-KD PARP2-KD
c
- LMB
HA-hPARP1 - + +
hHSF1-HA - + +
shRNA PARP13-KD - - +
SCR + + -
(kDa)
IB: anti-HSF1
IB: anti-PARP1
IB: anti-PARP13
IB: anti-actin
100 75 100 100 75 50 37
f
SCR HSF1-KD PARP1-KD PARP13-KD PARP2-KD
(kDa)
IB: anti-HSF1
IB: anti-PARP1
IB: anti-PARP13
IB: anti-PARP2
IB: anti-actin
75 150 100 100 75 75 50 37

Supplementary Fig. 2. PARP1 binds to DNA in a manner dependent on HSF1 and PARP13

(**a**) Cells, in which endogenous PARP1 was replaced with wild-type or its inactive mutant, were treated with DOX. Cell extracts were subjected to HSF1 immunoprecipitation and immunoblotting. (**b**) Dissociation of the ternary complex does not induce auto-PARylation of PARP1. HSF1 was substituted with each mutant or PARP13 was knocked down in HeLa cells. Some cells were treated with DOX. Denatured extracts of these cells were subjected to PARP1 immunoprecipitation, and then to immunoblotting using PAR antibody. Extracts were also subjected to immunoblotting using each antibody. (**c**) HeLa cells were infected with Ad-sh-SCR or Ad-sh-hPARP13-KD, and then with Ad-HA-hPARP1 and Ad-hHSF1. After being treated with LBM, extracts of these cells were subjected to immunoblotting using each antibody. PARP13 accumulated in the nucleus of LMB-treated cells (see Supplementary Fig. 1a). (**d**) ChIP-seq binding profiles of PARP1 at four arbitrarily chosen sites in HeLa cells treated as in **b**. ChIP-seq analysis was performed using scrambled RNA-treated cells (37 PARP1-binding peaks), LMB-treated cells overexpressing HA-hPARP1 and hHSF1-HA (744 peaks), and LMB-treated PARP13-knockdown cells overexpressing HA-hPARP1 and hHSF1-HA (246 peaks) in unstressed conditions. Gene names near the binding peaks and orientations are shown. Normalized read numbers and significantly enriched regions identified as peaks (red) are indicated. DNA regions amplified by qPCR are shown as red bars (peak regions) and black bars (negative control regions, NC) bars. Among genes near the 744 PARP1 binding peaks, the expression of 27 genes (3.6%) was altered in both HSF1 knockdown cells and cells expressing hHSF1-T20A (fold change > +1.3 or -1.3; $P < 0.05$) (see Supplementary Fig. 3c). (**e** and **f**) PARP1 binding in the absence of HSF1 and PARPs. ChIP assay of endogenous PARP1, PARP13, and HSF1 at the three sites as described above was performed in HeLa cells in which PARP1, PARP13, HSF1, and PARP2 were knocked down (n=3) (**e**). Cell extracts were subjected to immunoblotting (**f**). Mean ± s.d. is shown. Asterisks indicate $P < 0.01$ by Student's t test.

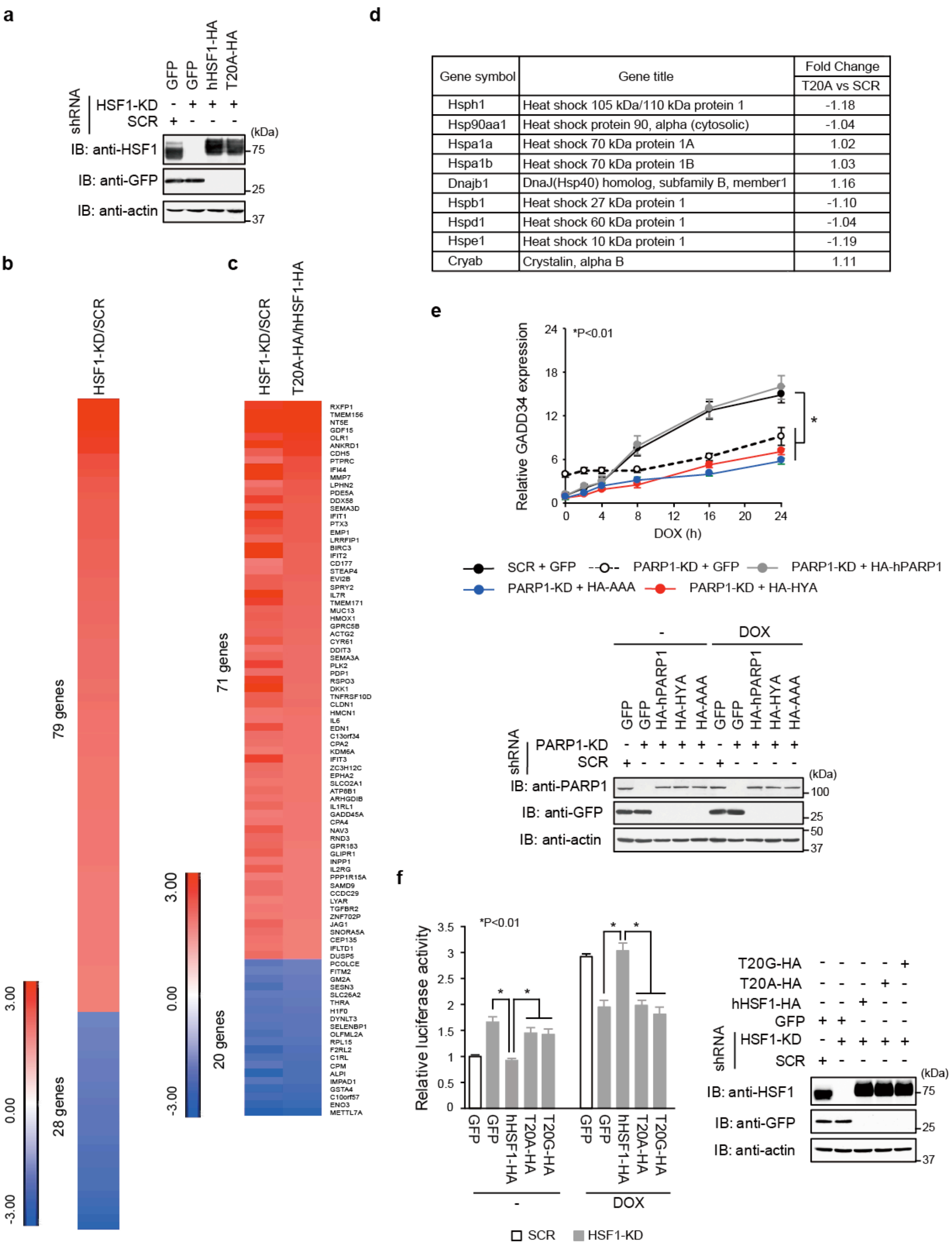

Gene symbol	Gene title	Fold Change
		T20A vs SCR
Hsph1	Heat shock 105 kDa/110 kDa protein 1	-1.18
Hsp90aa1	Heat shock protein 90, alpha (cytosolic)	-1.04
Hspa1a	Heat shock 70 kDa protein 1A	1.02
Hspa1b	Heat shock 70 kDa protein 1B	1.03
Dnajb1	DnaJ(Hsp40) homolog, subfamily B, member1	1.16
Hspb1	Heat shock 27 kDa protein 1	-1.10
Hspd1	Heat shock 60 kDa protein 1	-1.04
Hspe1	Heat shock 10 kDa protein 1	-1.19
Cryab	Crystalin, alpha B	1.11

Supplementary Fig. 3. HSF1-PARP13-PARP1 regulates the expression of DNA damage-inducible genes

(**a**) Protein levels of endogenous and ectopically expressed HSF1. Endogenous HSF1 was knocked down, and GFP, wild-type hHSF1 or hHSF1-T20A was reexpressed in HeLa cells. Scrambled RNA (SCR) was expressed as a control. Cell extracts were subjected to immunoblotting. (**b**) Heat map of differentially regulated genes in cells by HSF1 knockdown (fold change > +1.7 or -1.7; $P < 0.05$; n=3). (**c**) Heat map of differentially regulated genes by both HSF1 knockdown and substitution with hHSF1-T20A (fold change > +1.7 or -1.7; $P < 0.05$; n=3). Gene names are shown. (**d**) Fold changes of the expression of heat shock genes by the substitution with hHSF1-T20A. Basal expression levels in cells expressing hHSF1-T20A were compared with those in scrambled RNA-expressing cells (n=3). (**e**) Expression of the *GADD34* gene in cells expressing PARP1 mutants. Cells, in which endogenous PARP1 was replaced with each mutant, were treated with DOX for the indicated periods. GADD34 mRNA levels were quantified by RT-qPCR (upper). Extracts from cells before and after the treatment were subjected to immunoblotting (lower). Mean ± s.d. is shown. Asterisks indicate $P < 0.01$ by ANOVA. (**f**) Reporter analysis of the GADD34 promoter. HeLa-pGADD34-Luc cells, in which endogenous HSF1 was replaced with each protein, were treated with DOX for 16 h. Luciferase activity relative to that in untreated GFP-expressing cells is shown (upper). Extracts from cells before and after treatment were subjected to immunoblotting (lower). Mean ± s.d. is shown. Asterisks indicate $P < 0.01$ by Student's t test.

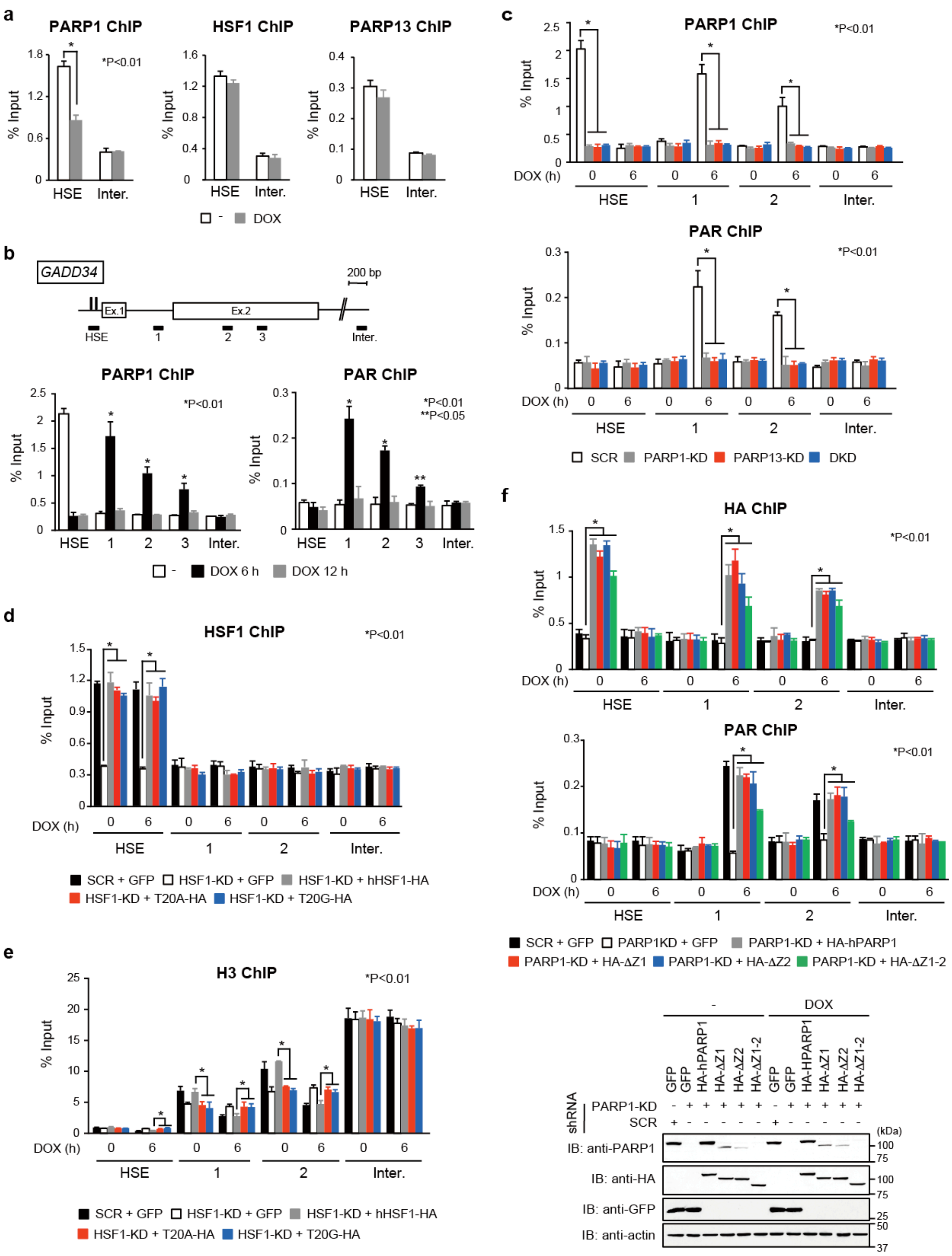
a
PARP1 ChIP
*P<0.01
HSF1 ChIP
PARP13 ChIP
% Input
HSE
Inter.
- DOX
b
GADD34
200 bp
Ex.1
Ex.2
HSE
1
2
3
Inter.
PARP1 ChIP
PAR ChIP
*P<0.01
**P<0.05
- DOX 6 h DOX 12 h
c
PARP1 ChIP
PAR ChIP
DOX (h)
SCR PARP1-KD PARP13-KD DKD
d
HSF1 ChIP
SCR + GFP HSF1-KD + GFP HSF1-KD + hHSF1-HA
HSF1-KD + T20A-HA HSF1-KD + T20G-HA
e
H3 ChIP
f
HA ChIP
PAR ChIP
SCR + GFP PARP1KD + GFP PARP1-KD + HA-hPARP1
PARP1-KD + HA-ΔZ1 PARP1-KD + HA-ΔZ2 PARP1-KD + HA-ΔZ1-2
-
DOX
GFP
HA-hPARP1
HA-ΔZ1
HA-ΔZ2
HA-ΔZ1-2
shRNA
PARP1-KD
SCR
(kDa)
IB: anti-PARP1
IB: anti-HA
IB: anti-GFP
IB: anti-actin
100
75
25
50
37

Supplementary Fig. 4. PARP1 redistribution and chromatin opening during DNA damage

(**a**) Occupancy of HSF1, PARP1, and PARP13 on the HSEs of the *GADD34* gene during DNA damage. ChIP-qPCR was performed after DOX treatment for 16 h (n=3). (**b**) Occupancy of PARP1 and PAR on the HSEs and *GADD34* gene loci during DOX treatment. ChIP-qPCR was performed 6 and 12 h after DOX treatment (n=3). Amplified DNA regions in the *GADD34* gene are shown in the diagram. (**c**) Occupancy of PARP1 and PAR on the *GADD34* locus in knockdown cells. ChIP-qPCR on the HSE and each gene locus (regions 1, 2 and 3) was performed before and after DOX treatment (n=3). Region 3 is located at the 3' end of the *GADD34* gene. Double knockdown (DKD) indicates knockdown of both PARP1 and PARP13. (**d**) Occupancy of HSF1 on the *GADD34* locus in cells expressing each hHSF1 mutant. ChIP-qPCR on the HSE and each gene locus (region 1 or 2) was performed before and after DOX treatment (n=3). (**e**) Occupancy of histone H3 on the *GADD34* locus in cells expressing each hHSF1 mutant. ChIP-qPCR was performed before and after DOX treatment (n=3). Mean ± s.d. is shown. Asterisks indicate $P < 0.01$ or o.05 by Student's t test. (**f**) Occupancy of PARP1 and PAR on the *GADD34* locus in cells expressing each HA-hPARP1 mutant. ChIP-qPCR on the HSE and each gene locus (region 1 or 2) was performed using HA or PAR antibody before and after DOX treatment (n=3) (upper). Mean ± s.d. is shown. Asterisks indicate $P < 0.01$ by Student's t test. Cell extracts were subjected to immunoblotting (lower).

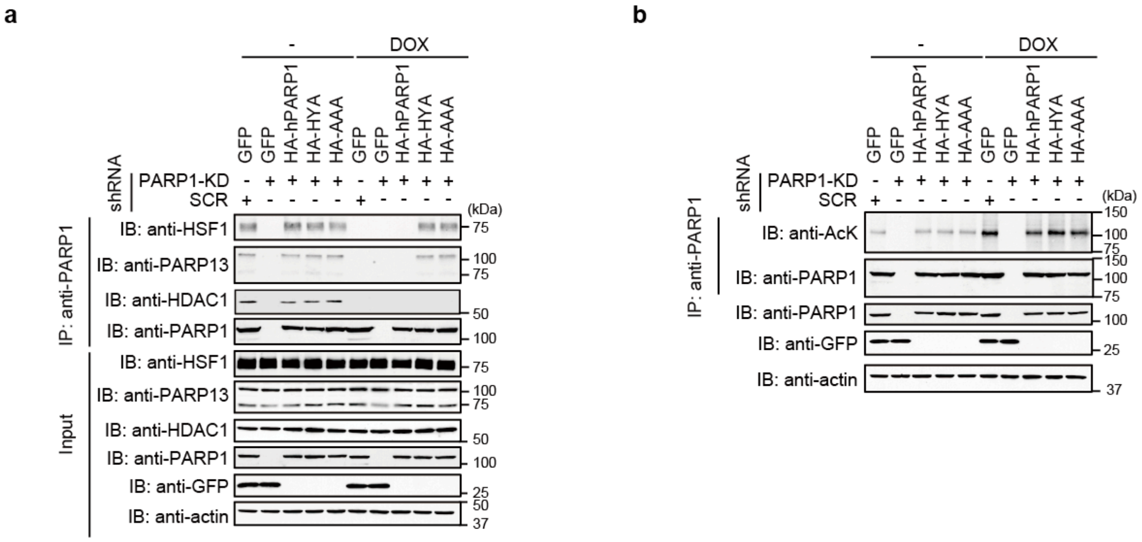

Supplementary Fig. 5. HDAC1 is released from inactive PARP1 mutants during DNA damage

(**a**) Cells, in which endogenous PARP1 was replaced with wild-type or its inactive hPARP1 mutant, were treated or not with DOX. Cell extracts were subjected to PARP1 immunoprecipitation and immunoblotting using antibodies including HDAC1 antibody. (**b**) Cells were treated as described in **a**. Denatured cell extracts were subjected to PARP1 immunoprecipitation and immunoblotting using antibodies including anti-acetyl lysine antibody (anti-AcK).

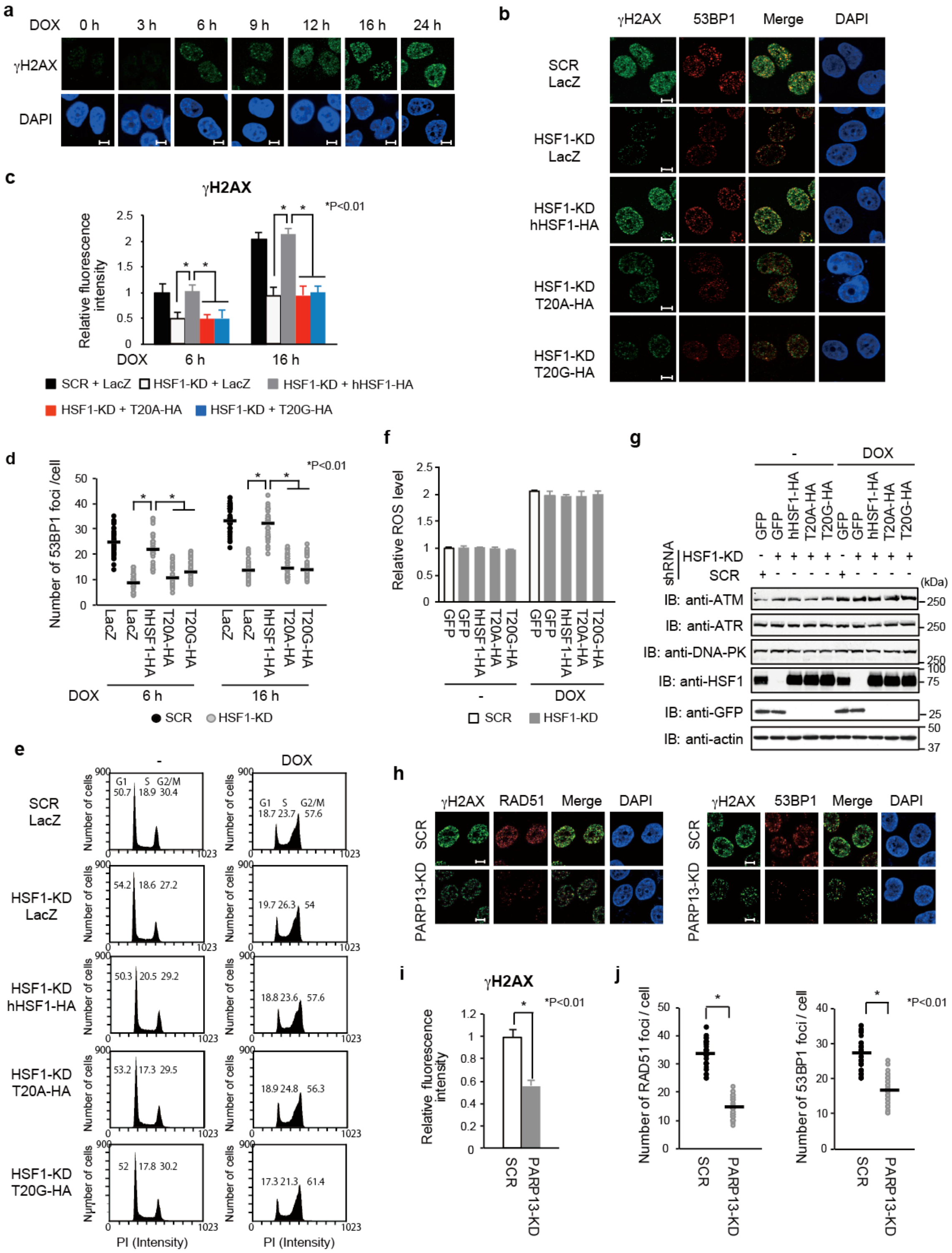
a
DOX 0 h 3 h 6 h 9 h 12 h 16 h 24 h
γH2AX
DAPI
b
γH2AX 53BP1 Merge DAPI
SCR LacZ
HSF1-KD LacZ
HSF1-KD hHSF1-HA
HSF1-KD T20A-HA
HSF1-KD T20G-HA
c
γH2AX
*P<0.01
Relative fluorescence intensity
DOX 6 h 16 h
SCR + LacZ
HSF1-KD + LacZ
HSF1-KD + hHSF1-HA
HSF1-KD + T20A-HA
HSF1-KD + T20G-HA
d
*P<0.01
Number of 53BP1 foci /cell
LacZ LacZ hHSF1-HA T20A-HA T20G-HA LacZ LacZ hHSF1-HA T20A-HA T20G-HA
DOX 6 h 16 h
SCR HSF1-KD
e
- DOX
SCR LacZ
HSF1-KD LacZ
HSF1-KD hHSF1-HA
HSF1-KD T20A-HA
HSF1-KD T20G-HA
Number of cells
G1 S G2/M
50.7 18.9 30.4
18.7 23.7 57.6
54.2 18.6 27.2
19.7 26.3 54
50.3 20.5 29.2
18.8 23.6 57.6
53.2 17.3 29.5
18.9 24.8 56.3
52 17.8 30.2
17.3 21.3 61.4
PI (Intensity)
f
Relative ROS level
GFP GFP hHSF1-HA T20A-HA T20G-HA GFP GFP hHSF1-HA T20A-HA T20G-HA
- DOX
SCR HSF1-KD
g
- DOX
GFP GFP hHSF1-HA T20A-HA T20G-HA GFP GFP hHSF1-HA T20A-HA T20G-HA
shRNA HSF1-KD
SCR
(kDa)
IB: anti-ATM 250
IB: anti-ATR 250
IB: anti-DNA-PK 250
IB: anti-HSF1 100 75
IB: anti-GFP 25
IB: anti-actin 50 37
h
γH2AX RAD51 Merge DAPI
γH2AX 53BP1 Merge DAPI
SCR
PARP13-KD
i
γH2AX
*P<0.01
Relative fluorescence intensity
SCR PARP13-KD
j
*P<0.01
Number of RAD51 foci / cell
Number of 53BP1 foci / cell
SCR PARP13-KD

Supplementary Fig. 6. HSF1 promotes DNA repair

(**a**) HeLa cells were treated with 0.5 μM DOX for the indicated periods, and stained with γH2AX antibody and DAPI. Fluorescence images were obtained using scanning confocal microscopy. Scale bar, 5 μm. (**b-d**) Cells, in which endogenous HSF1 was replaced with β-galactosidase (LacZ), wild-type hHSF1-HA, or its interaction mutants, were treated with DOX for 6 or 16 h, and then co-stained with γH2AX and 53BP1 antibodies and with DAPI. Fluorescence images were obtained using scanning confocal microscopy, and those including merged images (DOX for 16 h) are shown (**b**). Intensities of γH2AX fluorescence (**c**) and numbers of 53BP1 foci (**d**) in 50 cells were estimated. Mean ± s.d. is shown. Asterisks indicate $P < 0.01$ by Student's t test. (**e**) Cell cycle profiles. Cells, in which HSF1 was knocked down or replaced as described in **b**, were treated with DOX for 16 h, fixed, stained with propidium iodide, and analyzed with a flow cytometer (Cytomics FC500, Beckman Coulter). Numbers indicate percentages of cells in G1, S, and G2/M phases. (**f**) Production of reactive oxygen species (ROS) during DOX treatment. Cells, in which HSF1 was knocked down or replaced as described in **b**, were treated with DOX for 2 h, and ROS was measured with an ROS activity assay kit (Cell Meter Fluorimetric Intracellular Total ROS activity assay kit, Thermo Fisher Scientific) (n=3). (**g**) Extracts of cells treated as described in **f** were subjected to immunoblotting using ATM, ATR, and DNA-PK antibodies. (**h-j**) PARP13 knockdown cells (PARP13-KD) and control cells (SCR) were treated with DOX for 16 h, and then co-stained with γH2AX, RAD51 and 53BP1 antibodies and with DAPI. Fluorescence images including merged images are shown (**h**). Intensities of γH2AX fluorescence (**i**) and numbers of RAD51 and 53BP1 foci (**j**) in 50 cells were estimated. Mean ± s.d. is shown. Asterisks indicate $P < 0.01$ by Student's t test.

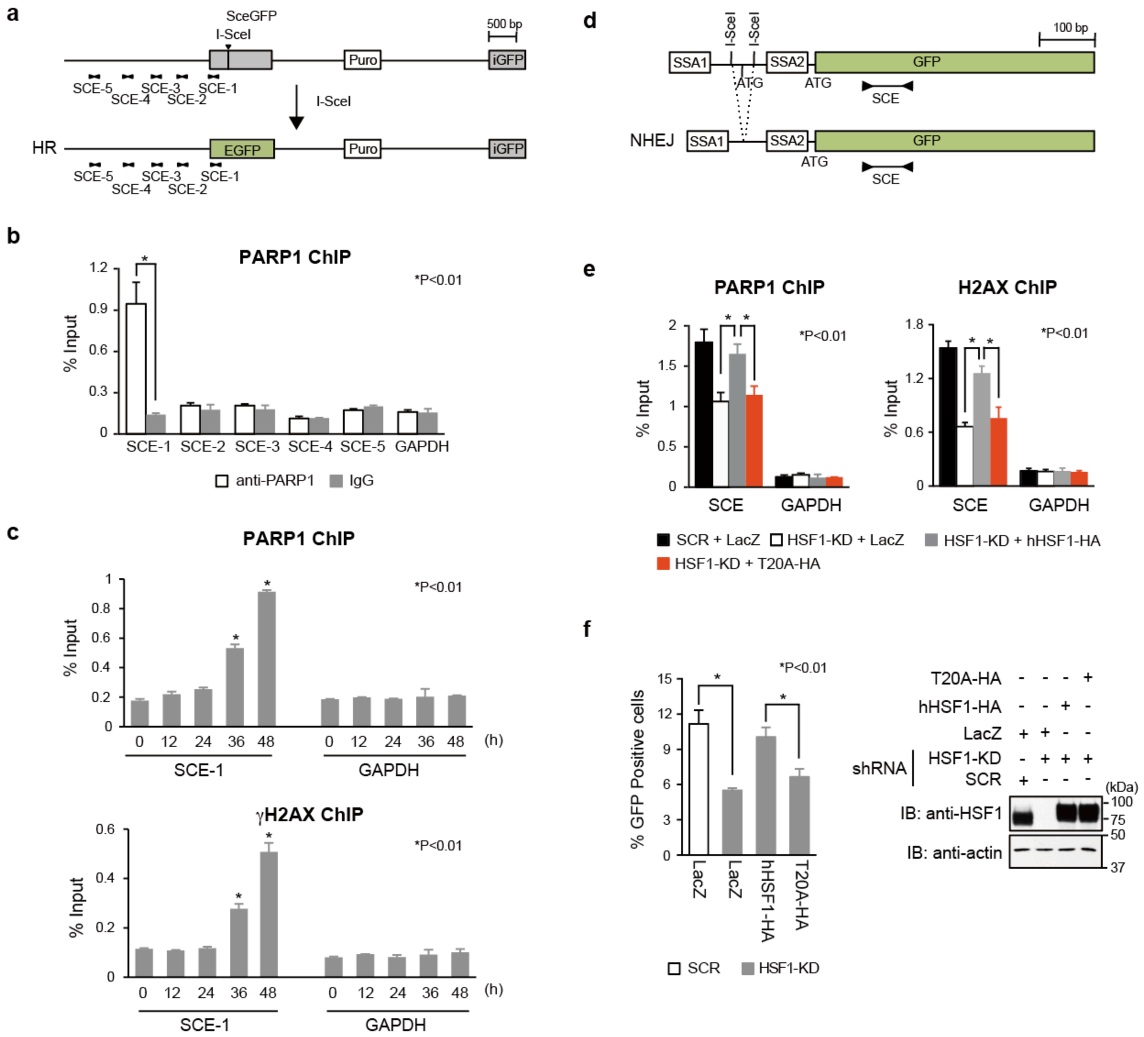
a
SceGFP
I-SceI
500 bp
Puro
iGFP
SCE-5
SCE-4
SCE-3
SCE-2
SCE-1
I-SceI
HR
EGFP
Puro
iGFP
SCE-5
SCE-4
SCE-3
SCE-2
SCE-1
b
PARP1 ChIP
*P<0.01
% Input
1.2
0.9
0.6
0.3
0
SCE-1 SCE-2 SCE-3 SCE-4 SCE-5 GAPDH
anti-PARP1
IgG
c
PARP1 ChIP
*P<0.01
% Input
1
0.8
0.6
0.4
0.2
0
0 12 24 36 48 0 12 24 36 48 (h)
SCE-1
GAPDH
γH2AX ChIP
*P<0.01
% Input
0.6
0.4
0.2
0
0 12 24 36 48 0 12 24 36 48 (h)
SCE-1
GAPDH
d
I-SceI
I-SceI
100 bp
SSA1
SSA2
GFP
ATG
ATG
SCE
NHEJ
SSA1
SSA2
GFP
ATG
SCE
e
PARP1 ChIP
*P<0.01
% Input
2
1.5
1
0.5
0
SCE GAPDH
H2AX ChIP
*P<0.01
% Input
1.8
1.2
0.6
0
SCE GAPDH
SCR + LacZ
HSF1-KD + LacZ
HSF1-KD + hHSF1-HA
HSF1-KD + T20A-HA
f
*P<0.01
% GFP Positive cells
15
12
9
6
3
0
LacZ
LacZ
hHSF1-HA
T20A-HA
SCR
HSF1-KD
T20A-HA - - - +
hHSF1-HA - - + -
LacZ + + - -
shRNA
HSF1-KD - + + +
SCR + - - -
(kDa)
100
75
50
37
IB: anti-HSF1
IB: anti-actin

Supplementary Fig. 7. HSF1 promotes the redistribution of PARP1 during repair of DSB by HRR and NHEJ

(**a**) Schematic showing amplified regions in pDR-GFP before and after HR repair. SCE-1 to SCE-5 regions amplified by ChIP-qPCR is upstream of the I-SceI cutting site. A complete *EGFP* gene was generated by HRR of the I-Sce-mediated DSB. (**b**) Accumulation of PARP1 in five SCE regions. ChIP analysis was performed using cells transfected with pCBASce for 48 h (**c**) Accumulation of PARP1 and γH2AX in a SCE-1 region at different time points. ChIP analysis was performed using cells transfected with pCBASce for the indicated periods (n=3). Mean ± s.d. is shown. Asterisks indicate $P < 0.01$ by Student's t test. (**d**) Schematic showing an amplified region SCE in the pEJSSA reporter construct before and after NHEJ. The I-SceI cutting sites and translational start sites (ATG) are shown. Repair of the I-SceI-induced DSB by NHEJ restores GFP translation. (**e**) Accumulation of PARP1 and γH2AX in the SCE region. ChIP assay was performed using cells, in which endogenous HSF1 was replaced with β-galactosidase (LacZ), hHSF1-HA, or hHSF1-T20A-HA (n=3). Mean ± s.d. is shown. Asterisks indicate $P < 0.01$ by Student's t test. (**f**) Efficiency of NHEJ in cells expressing an HSF1 interaction mutant. Numbers of GFP-positive cells from 200 cells were counted, and the percentage of these cells is shown (n=3) (left). Mean ± s.d. is shown. Asterisks indicate $P < 0.01$ by Student's t test. HSF1 levels were examined by immunoblotting (right).

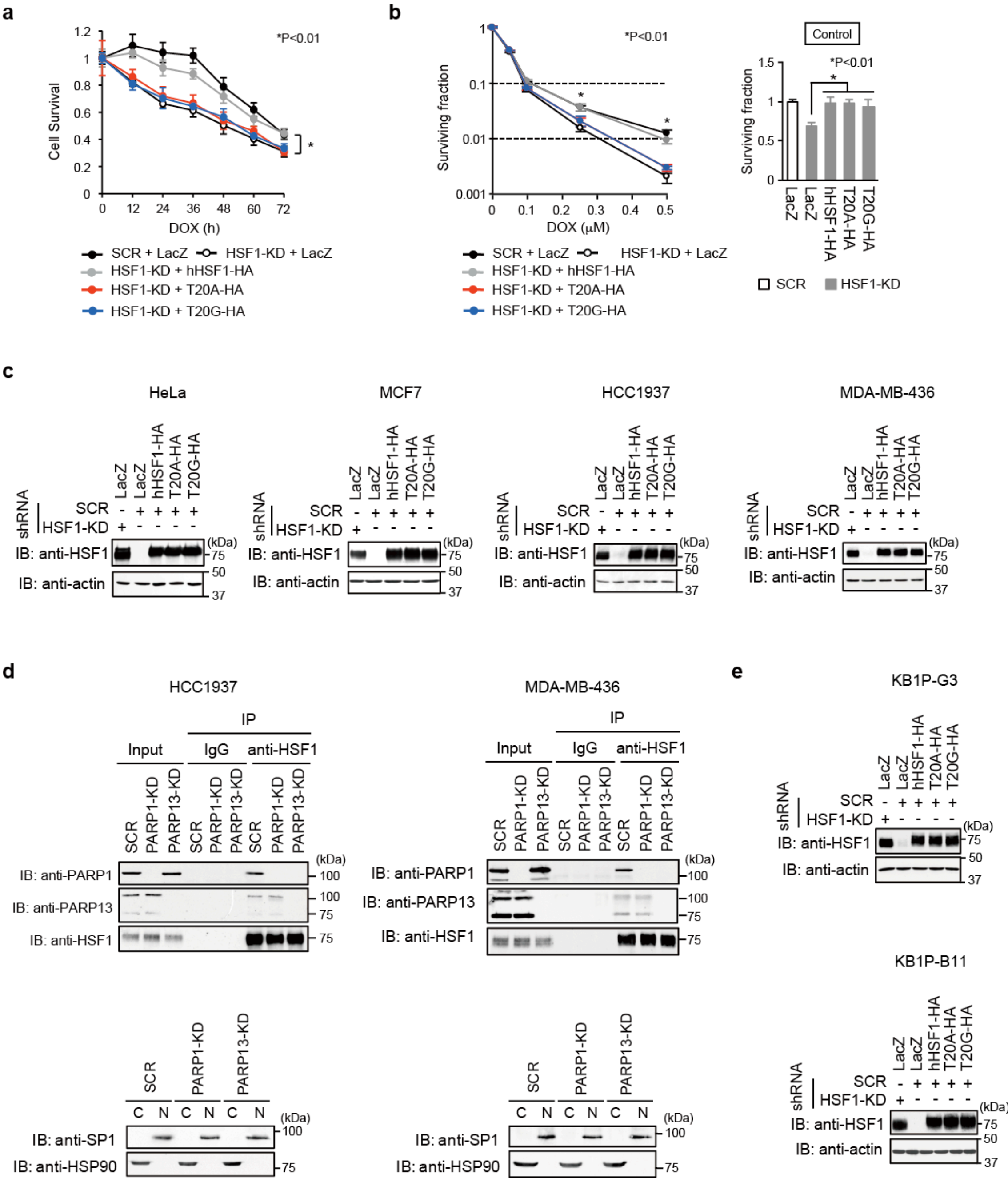
a
Cell Survival
DOX (h)
*P<0.01
SCR + LacZ
HSF1-KD + LacZ
HSF1-KD + hHSF1-HA
HSF1-KD + T20A-HA
HSF1-KD + T20G-HA
b
Surviving fraction
DOX (μM)
*P<0.01
Control
SCR
HSF1-KD
c
HeLa
MCF7
HCC1937
MDA-MB-436
shRNA
SCR
HSF1-KD
LacZ
hHSF1-HA
T20A-HA
T20G-HA
IB: anti-HSF1
IB: anti-actin
(kDa)
d
HCC1937
MDA-MB-436
Input
IP
IgG
anti-HSF1
SCR
PARP1-KD
PARP13-KD
IB: anti-PARP1
IB: anti-PARP13
IB: anti-HSF1
C
N
IB: anti-SP1
IB: anti-HSP90
e
KB1P-G3
KB1P-B11

Supplementary Fig. 8. HSF1-PARP13-PARP1 protects cells from genotoxic stress

(**a**) Reduced survival of DOX-treated HeLa cells expressing hHSF1-T20A or hHSF1-T20G. Cells, in which endogenous HSF1 was replaced with β-galactosidase (LacZ), hHSF1-HA, or its interaction mutants, were treated with 0.5 μM DOX. Relative cell numbers at each time point are shown (n=3). Mean ± s.d. is shown. Asterisks indicate $P < 0.01$ by ANOVA. (**b**) Reduced clonogenic survival of DOX-treated HeLa cells expressing hHSF1-T20A or hHSF1-T20G. Cells described in **a** were treated with DOX (0, 0.05, 0.1, 0.25, or 0.5 μM) for 16 h. Colony numbers relative to that of DOX-untreated, scrambled RNA-treated cells are shown as a surviving fraction (n=3) (left). Colony numbers of DOX-untreated cells are also shown (n=3) (right). Mean ± s.d. is shown. Asterisks indicate $P < 0.01$ by Student's t test. (**c**) Endogenous HSF1 was replaced with β-galactosidase (LacZ), hHSF1-HA, or its interaction mutants in human HeLa and three mammary tumor cells. HSF1 levels were examined by immunoblotting. (**d**) Immunoprecipitation in nuclear fractions of HCC1937 and MDA-MB-436 cells, in which PARP1 or PARP13 was knocked down by infection with adenovirus expressing the corresponding shRNA, or scrambled RNA (SCR) as control. Nuclear (N) and cytoplasmic (C) fractions were blotted using an antibody for nuclear (SP1) or cytoplasmic (HSP90) protein. (**e**) Endogenous HSF1 was replaced with β-galactosidase (LacZ), hHSF1-HA, or its interaction mutants in mouse mammary tumor KB1P-G3 and KB1P-B11 cells. HSF1 levels were examined by immunoblotting.

Fig. 1a

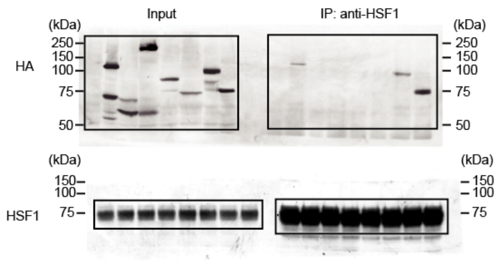

Fig. 1b

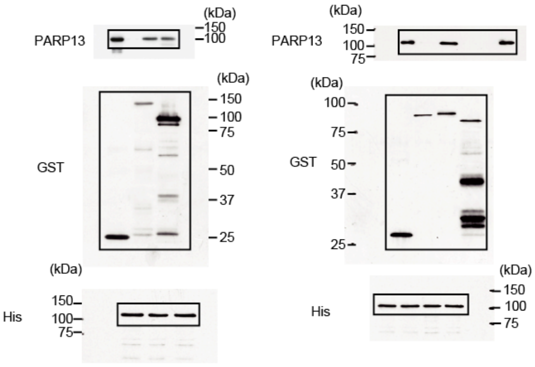

Fig. 1c

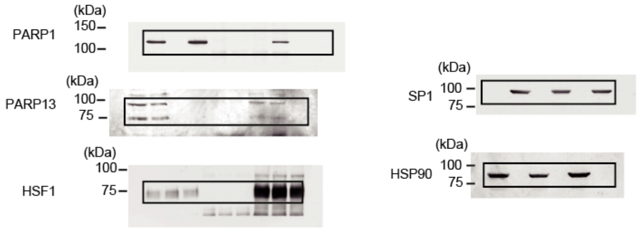

Fig. 1d

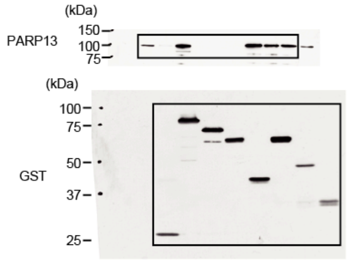

Fig. 1f

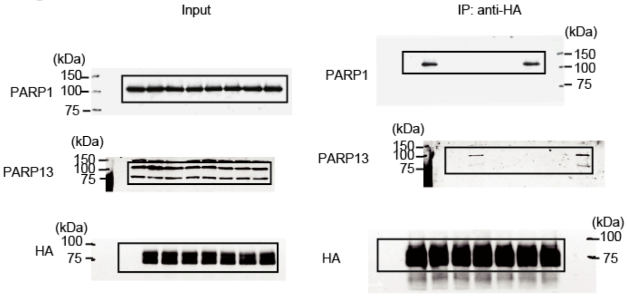

Fig. 1g

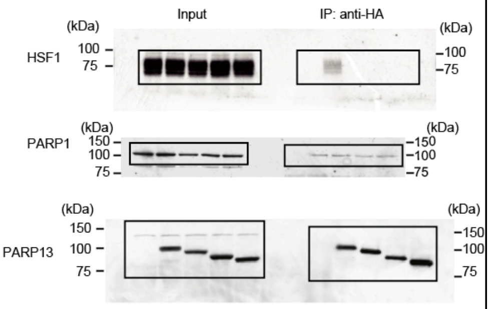

Fig. 2a

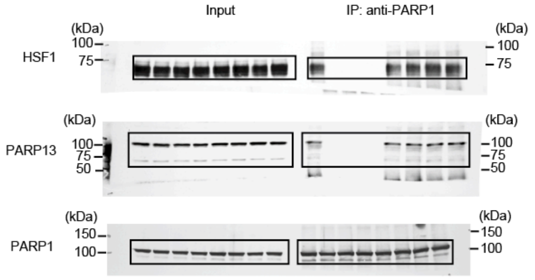

Fig. 2b

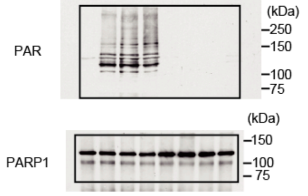

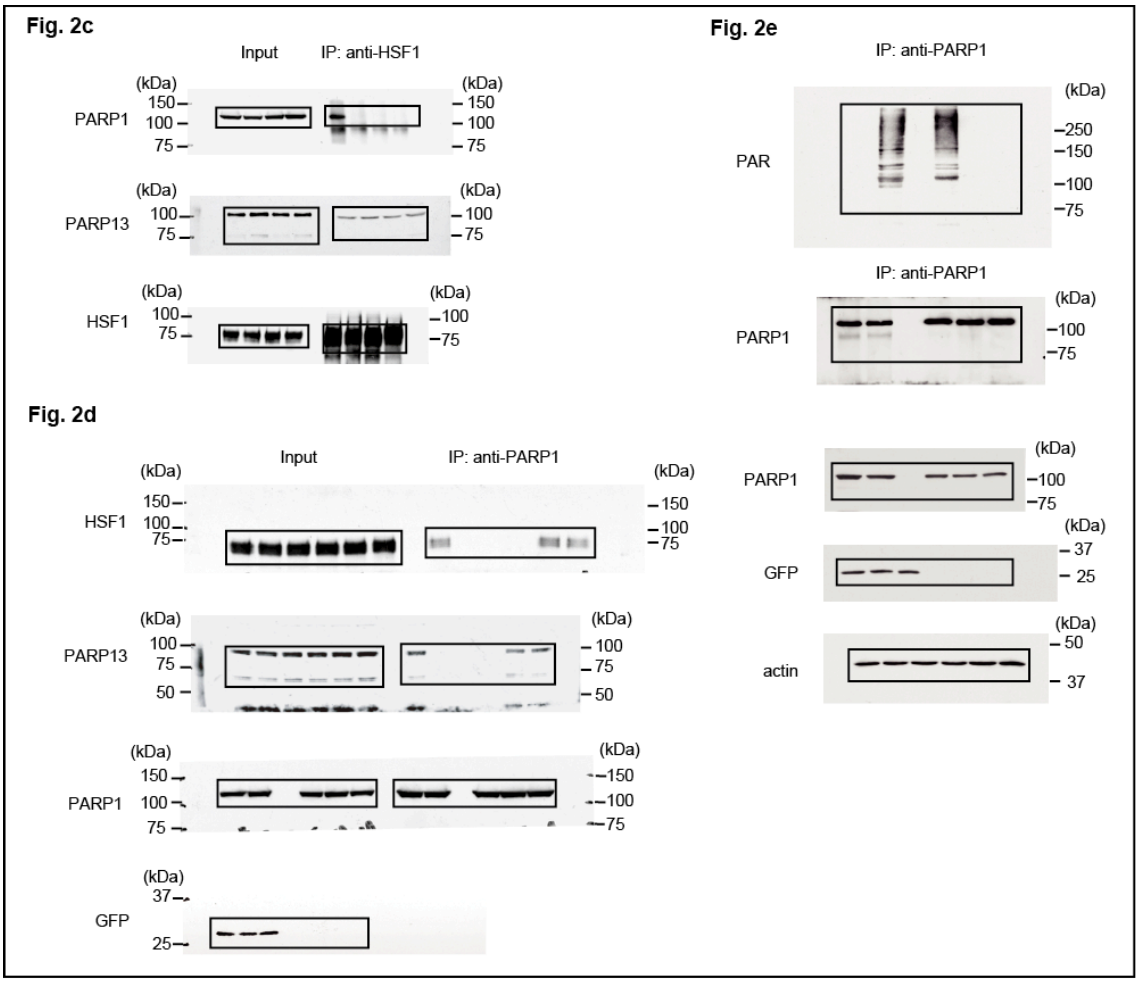
Fig. 2c
Input
IP: anti-HSF1
(kDa)
PARP1
150
100
75
PARP13
HSF1
Fig. 2d
IP: anti-PARP1
GFP
37
25
50
Fig. 2e
PAR
250
actin

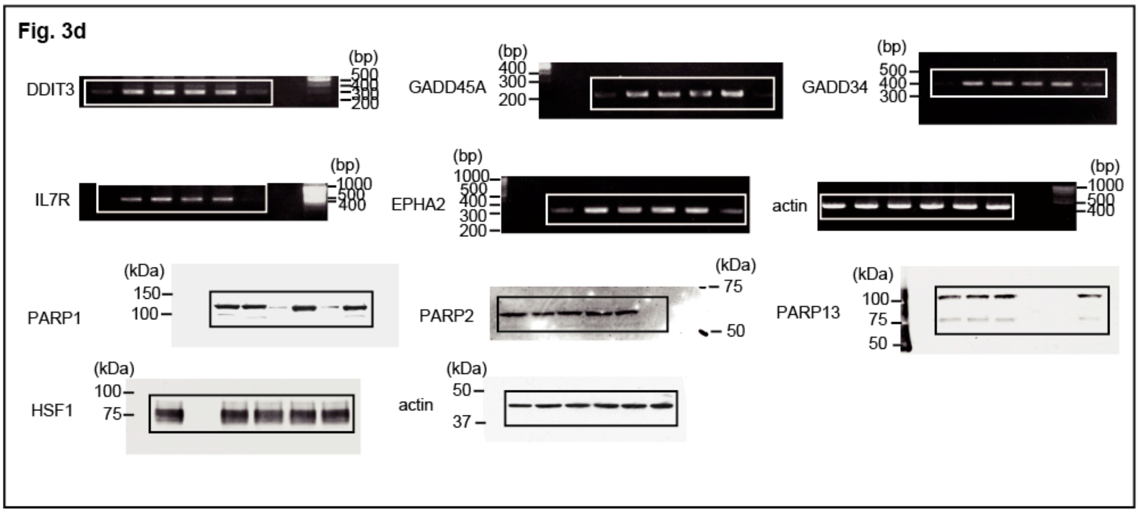
Fig. 3d
(bp)
DDIT3
GADD45A
GADD34
IL7R
EPHA2
actin
1000
500
400
300
200
(kDa)
PARP1
PARP2
PARP13
HSF1
150
100
75
50
37

Fig. 3e

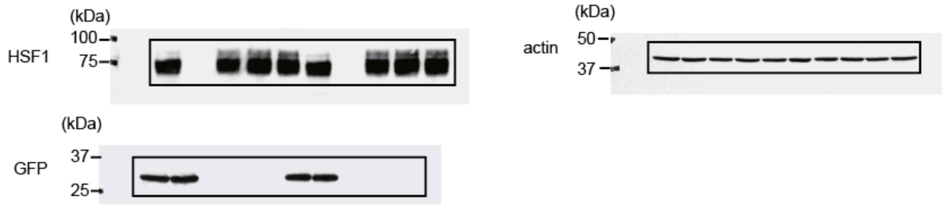

Fig. 3f

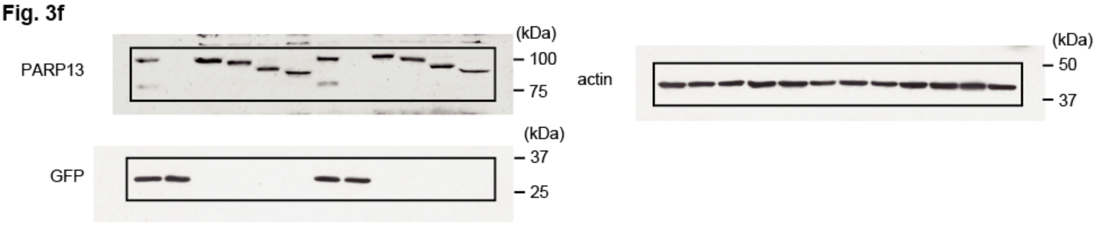

Fig. 5a

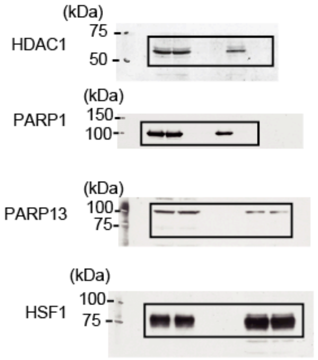

Fig. 5c

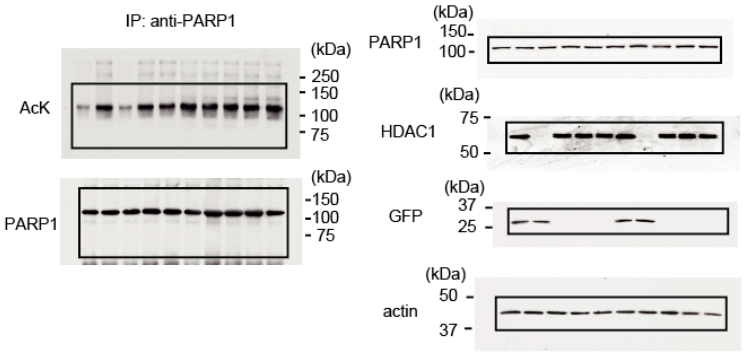

Fig. 5d

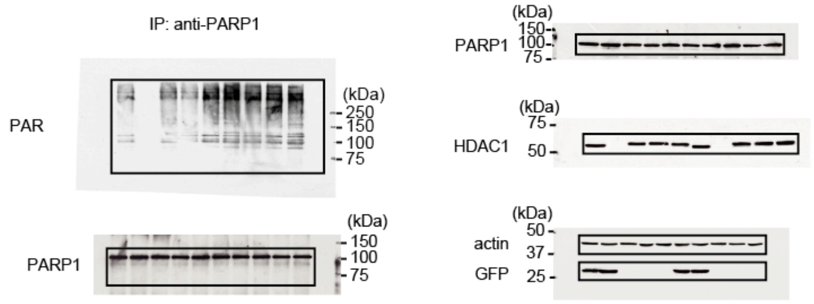

Fig. 5e

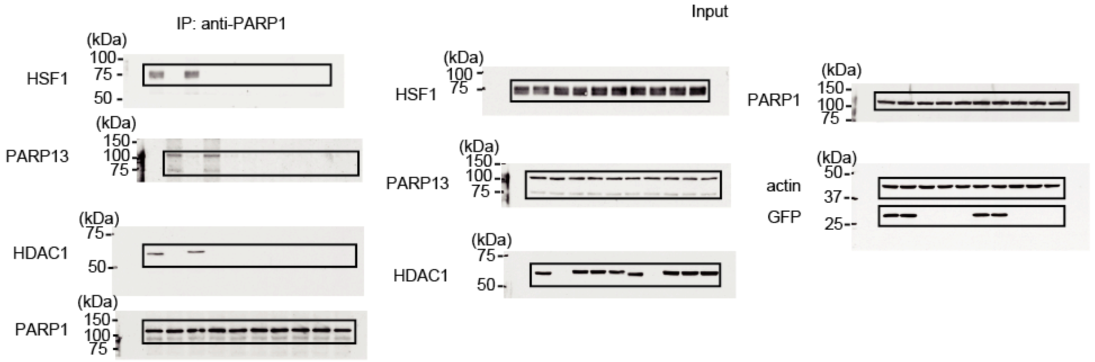

Fig. 5e

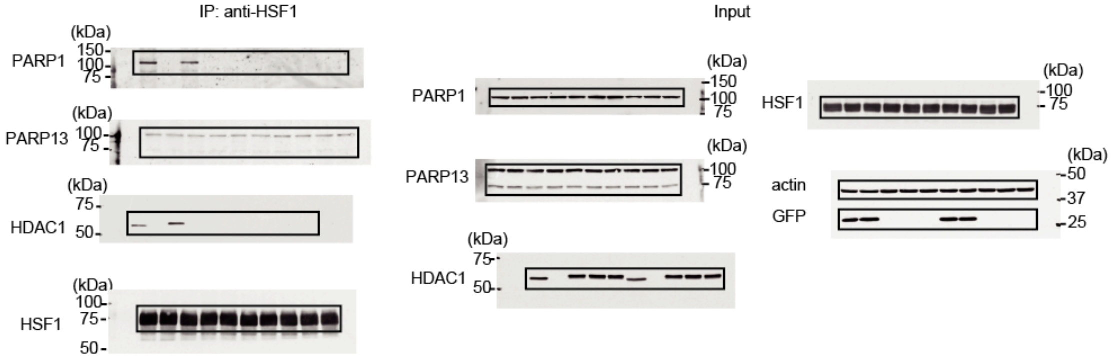

Fig. 6g

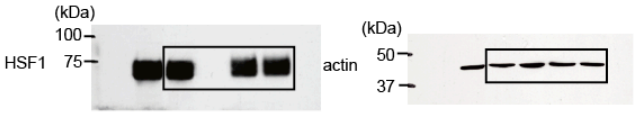

Supplementary Fig. 1b

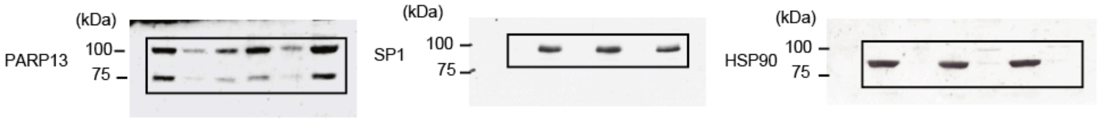

Supplementary Fig. 1c

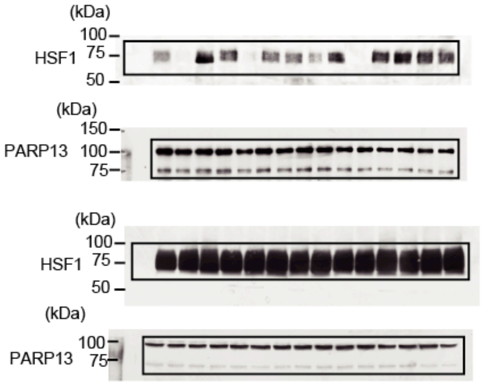

Supplementary Fig. 1d

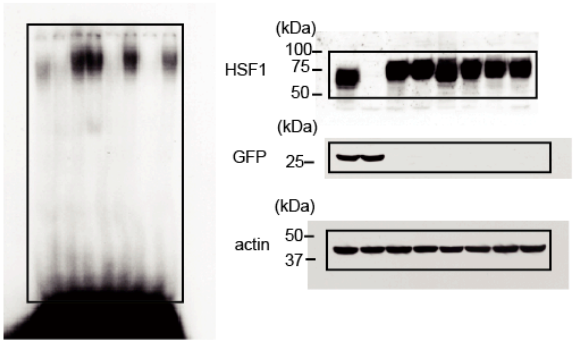

Supplementary Fig. 1f

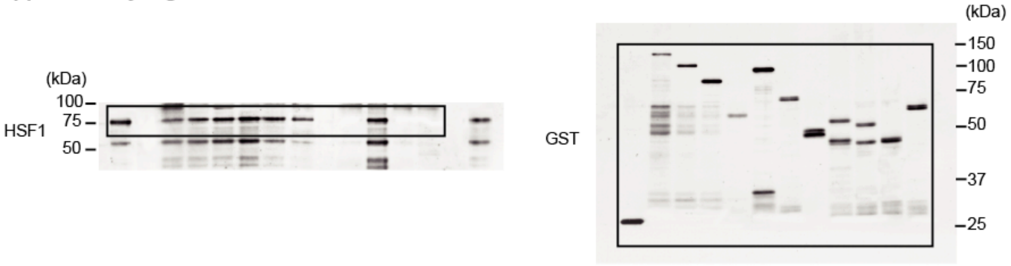

Supplementary Fig. 2a

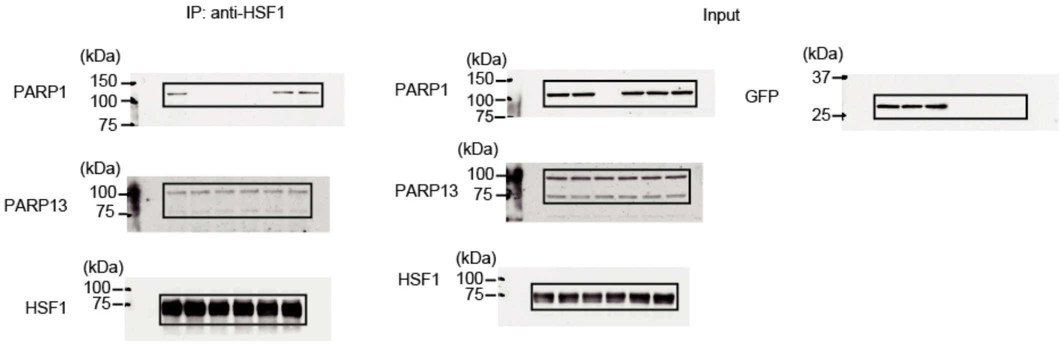

Supplementary Fig. 2b

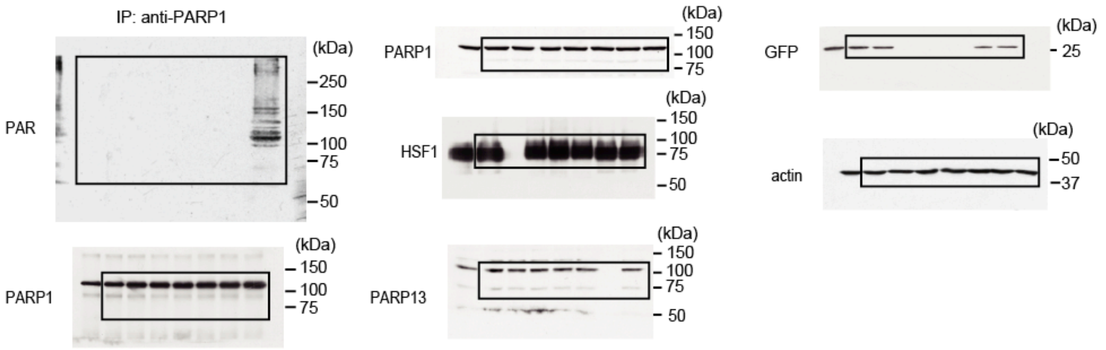

Supplementary Fig. 2c

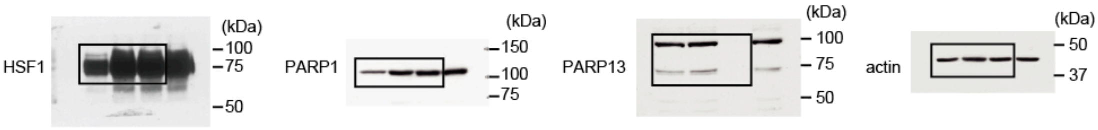

Supplementary Fig. 2f

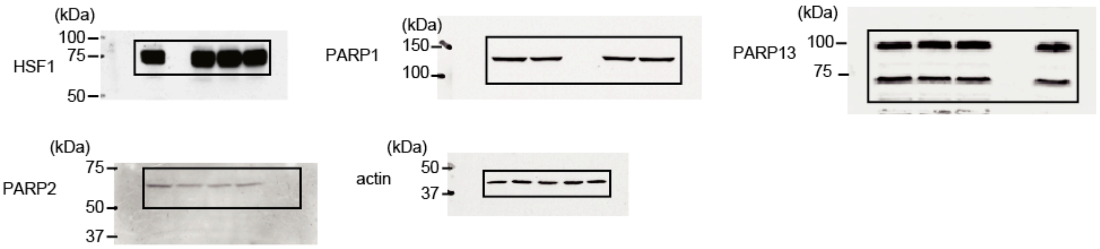

Supplementary Fig. 3a

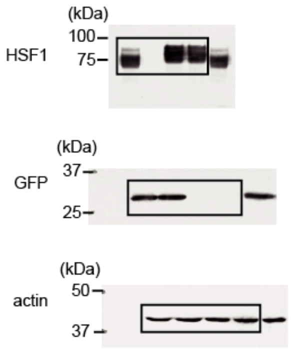

Supplementary Fig. 3e

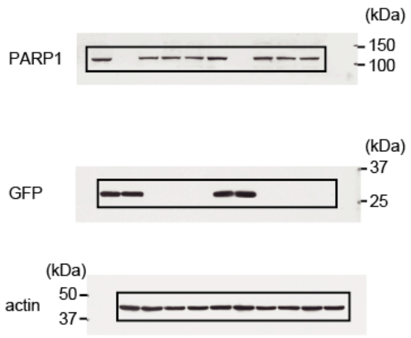

Supplementary Fig. 3f

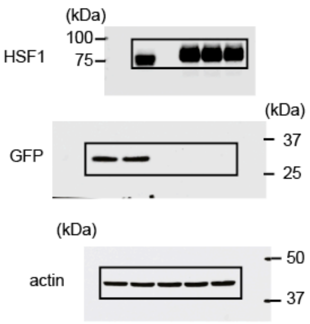

Supplementary Fig. 4f

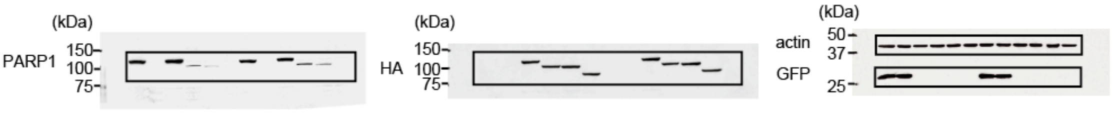

Supplementary Fig. 5a

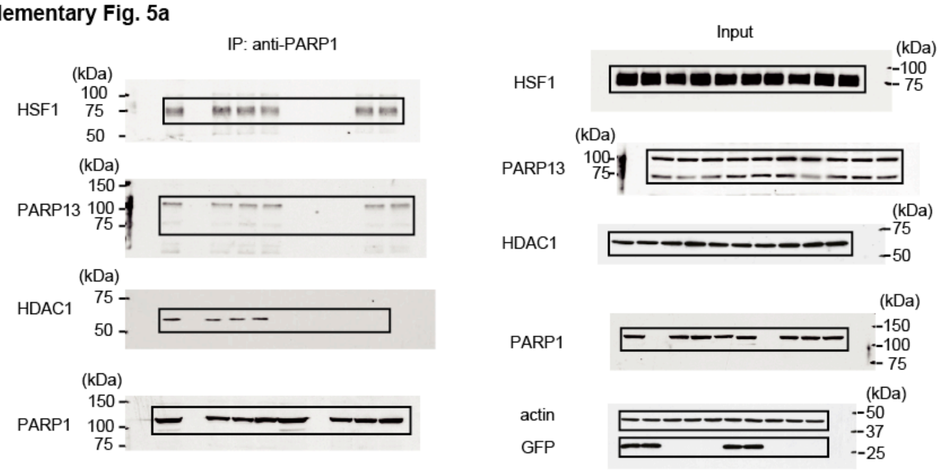

Supplementary Fig. 5b

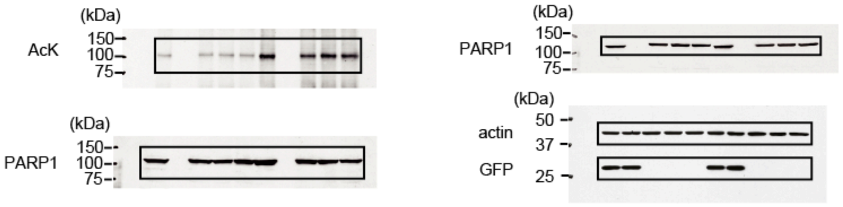

Supplementary Fig. 6g

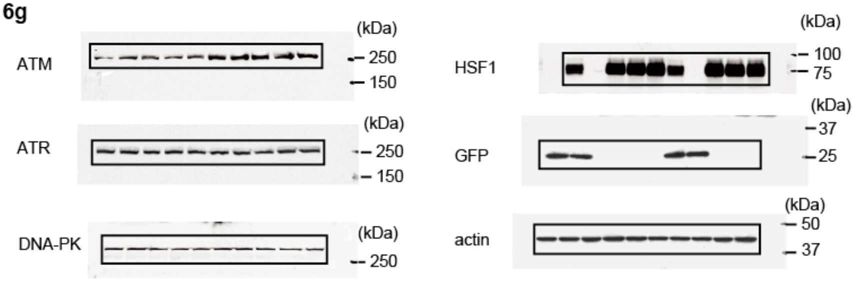

Supplementary Fig. 7f

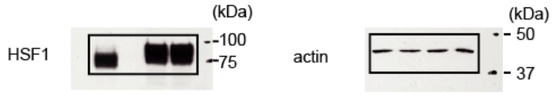

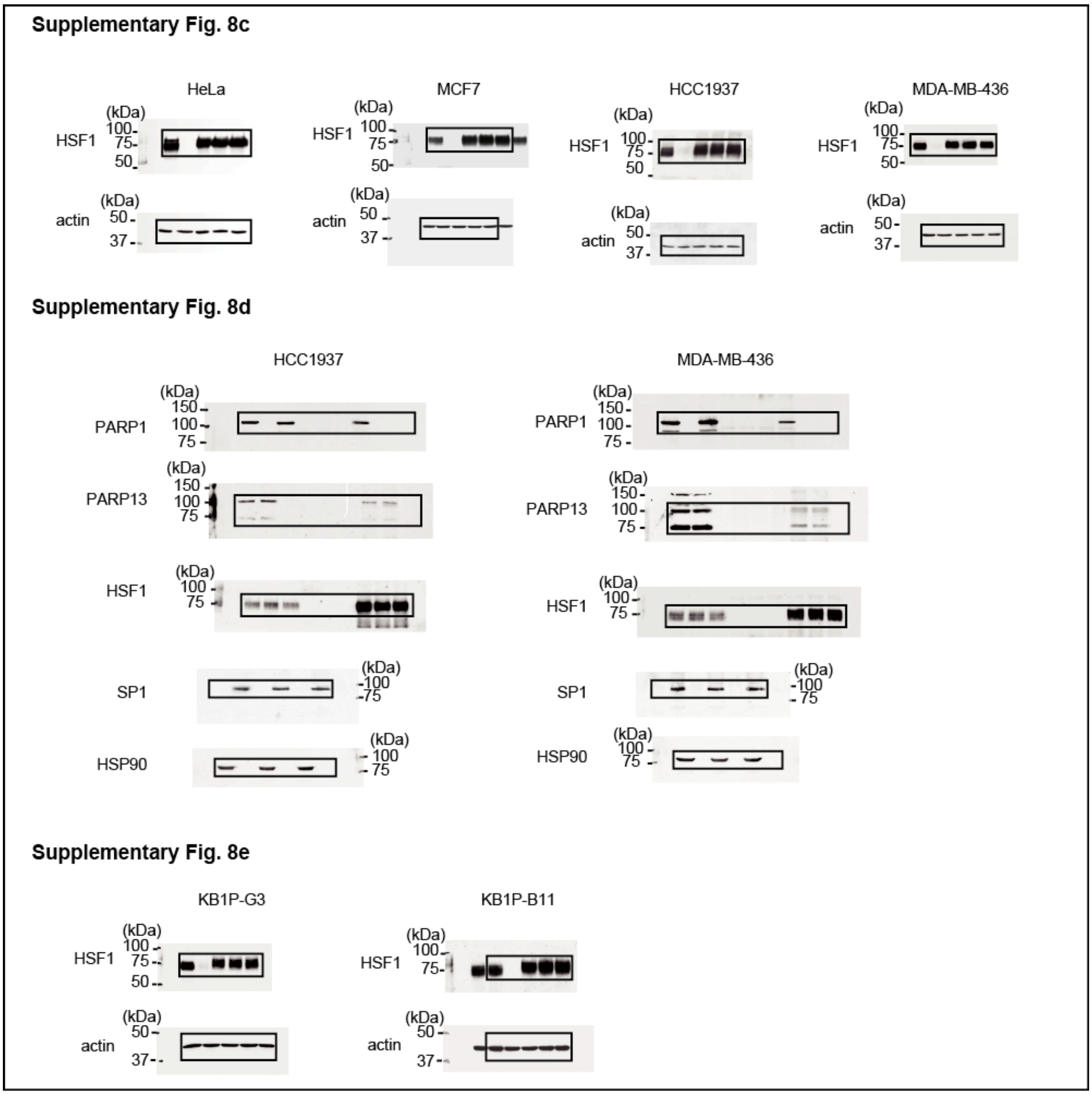

Supplementary Fig. 9. Uncropped scans of Western blots and gels of DNA and EMSA.

Supplementary Table 1. Primer sequences used for knockdown

shRNA	Sense strand	Antisense Strand
SCR	5'-GATCCATGTACTGCGCGTGGAGACTTCAAGA GAGTCTCCACGCGCAGTACATTCTTTTTTGGAAA-3'	5'-AGCTTTTCCAAAAAAAAGAGCTATGAAGATTCG ATTTCTCTTGAAGTCTCCACGCGCAGTACATG-3'
hPARP1-KD	5'-GATCCGATAGAGCGTGAAGGCGAACTCAAGAGA TTCGCCTTCACGCTCTATCTTTTTTGGAAA-3'	5'-AGCTTTTCCAAAAAAGATAGAGCGTGAAGGCG AA TCTCTTGAG TTCGCCTTCACGCTCTATCG-3'
hPARP2-KD	5'-GATCCGAGAGAAGAACAGAAATAGTTCAAGAGA CTATTTCTGTTCTTCTCTCTTTTTTGGAAA-3'	5'-AGCTTTTCCAAAAAAGAGAGAAGAACAGAAATA GTCTCTTGAACTATTTCTGTTCTTCTCTCG-3'
hPARP13-KD	5'-GATCCGAGTAGCACTTGTTAACGATTCAAGAGA TCGTTAACAAGTGCTACTCTTTTTTGGAAA-3'	5'-AGCTTTTCCAAAAAAGAGTAGCACTTGTTAACG ATCTCTTGAATCGTTAACAAGTGCTACTCG-3'
hHDAC1-KD	5'-GATCCTTCTTAACTTTGAACCATATTCAAGAGA TATGGTTCAAAGTTAAGAACGTTTTTTGGAAA-3'	5'-AGCTTTTCCAAAAAACGTTCTTAACTTTGAACC ATATCTCTTGAATATGGTTCAAAGTTAAGAAG-3'

Supplementary Table 2. RT-PCR primer sequences

RT-PCR	Forward primer	Reverse Primer
DDIT3	5'-AGAGCTGGAACCTGAGGAGAGA-3'	5'-AGCAGGGTCAAGAGTGGTGAAG-3'
GADD45A	5'-ATTAATCTCCCTGAACGGTGATG-3'	5'-GCTTCCTTCTTCATTTTCACCTCT-3'
GADD34	5'-AGCTGAGTGTCCTCCCTGCATC-3'	5'-GGGTCTGACTCAGCTTCTCCCAA-3'
IL7R	5'-GGTAAATTACCCAACCTCACACGTT-3'	5'-GAAGAGGCAAAGGCTGGTAGGA-3'
EPHA2	5'-AGCCTTCGGACAGACATATGGGA-3’	5'-GCATCTTGCAAAGGCCCAGGAA-3’
hactin	5'-GTGCCCATCTACGAGGGGTATG-3’	5'-GACTTCCACACTCACACAGTAAGTAAGC-3’

Supplementary Table 3. RT-qPCR primer sequences

qRT-PCR	Forward primer	Reverse Primer
GADD45A	5'-GGAGGAAGAGAATCAAGCCA-3'	5'-TGGGGTCGGAGCCTGAAGAT-3'
GADD34	5'-AGTCAGCGCACGATCACTGT-3'	5'-GGATCAGGGTGAAGTGGATCTG-3'
hactin	5'-AGAGCTACGAGCTGCCTGAC-3'	5'-AGCACTGTGTTGGCGTACAG-3'

Supplementary Table 4. ChIP-qPCR primer sequences

CHIP-qPCR	Forward primer	Reverse Primer
BCL11A	5'-GAAGATTGGGTTGGAAGGACAC-3'	5'-GATAGCAGAAAAGTAGCATTTA-3'
BCL11A-Inter	5'-GGCTACCATGCAGGATTTGAATT-3'	5'-GATTTACCGCTCGCCCAGTGAT-3'
TMCC3	5'-AAGACTGCCCTTCAAATGCC-3'	5'-GAGTTCTGTGTGCTGCTCT-3'
TMCC3-Inter	5'-GGCACATGTCTCAGGCAGAGTG-3'	5'-GTTCTGAAAAGGAGCACAGACT-3'
ST6GALNAC5	5’-GAGCAGACCACTCCCAGCCCAA-3'	5’-GTTGCAAGTAACAGGGGCCTAT-3'
ST6GALNAC5-Inter	5’-GCATTAACAAGGAGCCAGTTCTG-3'	5’-GCCTCTTGACCTTTTGACCACA-3'
KDM4A	5’-GTGAGATTTGGTTGTAGCTTTGG-3'	5’-GATGAGCAAATTAAACCCAGTG-3'
KDM4A-Inter	5’-TGAACTGCCAGTGAGTGCTTAT-3'	5’-GCTACTAGAGTGCTGGATGAGA-3'
GADD34-HSE	5'-AGTCAGCGCACGATCACTGT-3'	5'-GCTGACGTCACGAAGAGAGGCG-3'
GADD34-1	5'-GACCCTTGGATCTAAGG-3'	5'-CTCAATAGCCAGGAGTCT-3'
GADD34-2	5'-CCTGTCTCCCAGCCTTCTGA-3'	5'-TTTCTCCTCCCCTGGGTTCTT-3'
GADD34-3	5’-CAGAGGCCCCAGCTCAAG-3’	5’-GCAGGGAGGACACTCAGCT-3’
GADD34-Inter	5'-GAGTAATTGCTACAAACTAATGA-3'	5'-GAATAATTATACCAGATATAGA-3'
HR-SCE-1	5'-TCATTTTGGCAAAGAATTCAGATC-3'	5'-TTCTTCGGCACCTTTCTCTTC-3'
HR-SCE-2	5'-GAGGCGCGGCGAGCCGCAG-3'	5'-ATTTGGGACAAAGGAAGTCC-3'
HR-SCE-3	5'-GTTTAATGACGGCTCGTTTC-3'	5'-TCCCGGAGCCCTTTAAGGCT-3'
HR-SCE-4	5’-GACTTTCCTACTTGGCAGTA-3’	5’-GCTCACCTCGACCCATGGTAAT-3’
HR-SCE-5	5’-CATGAGCGGATACATATTTGAATGT-3’	5’-GTCGACCAGGTGGCACTTTT-3’
NHEJ-SCE	5’-GACGTAAACGGCCACAAGTT-3’	5’-ATGAACTTCAGGGTCAGCTT-3’
LUC	5’-CGCATGCCAGAGATCCTATT-3’	5’-CAAAACCGTGATGGAATGGAA-3’
GAPDH	5’-TCTCCCCACACACATGCACTT-3’	5’-CCTAGTCCCAGGGCTTTGATT-3’
